# The association between e-cigarette use and asthma among never combustible cigarette smokers: behavioral risk factor surveillance system (BRFSS) 2016 & 2017

**DOI:** 10.1186/s12890-019-0950-3

**Published:** 2019-10-16

**Authors:** Albert D. Osei, Mohammadhassan Mirbolouk, Olusola A. Orimoloye, Omar Dzaye, S. M. Iftekhar Uddin, Zeina A. Dardari, Andrew P. DeFilippis, Aruni Bhatnagar, Michael J. Blaha

**Affiliations:** 1 The American Heart Association Tobacco Regulation and Addiction Center, Dallas, TX USA; 2 Johns Hopkins University, Carnegie 583 JHH, 600 N Wolfe St, Baltimore, MD 21287 USA; 3 Department of Radiology and Neuroradiology, Charité, Berlin, Germany; 4 University of Louisville, Louisville, KY USA

**Keywords:** Asthma, E-cigarettes, Combustible cigarettes

## Abstract

**Background:**

E-cigarette use prevalence has grown rapidly in the US. Despite the popularity of these products, few acute exposure toxicity studies exist, and studies on long-term pulmonary health effects are limited. E-cigarette users who are never combustible cigarette smokers (sole users) constitute a unique group of young adults that may be at increased risk of bronchial hyperreactivity and development of asthma. Given the public health concern about the potential pulmonary health effects of sole e-cigarette use, we aimed to examine the association between e-cigarette use and asthma among never combustible cigarette smokers.

**Methods:**

We pooled 2016 and 2017 data of the Behavioral Risk Factor Surveillance System (BRFSS), a large, cross-sectional telephone survey of adults aged 18 years and older in the U.S. We included 402,822 participants without any history of combustible cigarette smoking (defined as lifetime smoking < 100 cigarettes) and with complete self-reported information on key variables. Current e-cigarette use, further classified as daily or occasional use, was the primary exposure. The main outcome, asthma, was defined as self-reported history of asthma. We assess the relationship of sole e-cigarette use with asthma using multivariable logistic regression adjusting for age, sex, race, income, level of education and body mass index.

**Results:**

Of 402,822 never combustible cigarette smokers, there were 3103 (0.8%) current e-cigarette users and 34,074 (8.5%) with asthma. The median age group of current e-cigarette users was 18–24 years. Current e-cigarette use was associated with 39% higher odds of self-reported asthma compared to never e-cigarette users (Odds Ratio [OR], 1.39; 95% confidence interval: 1.15, 1.68). There was a graded increased odds of having asthma with increase of e-cigarette use intensity. The odds ratio of self-reported asthma increased from 1.31 (95% confidence interval: 1.05, 1.62) in occasional users to 1.73 (95% confidence interval: 1.21, 2.48) in daily e-cigarette users, compared to never e-cigarette users.

**Conclusion:**

Our findings from a large, nationally representative survey suggest increased odds of asthma among never combustible smoking e-cigarette users. This may have potential public health implications, providing a strong rationale to support future longitudinal studies of pulmonary health in young e-cigarette-using adults.

## Background

In 2016, 10.8 million U.S. adults reported current e-cigarette use [1] of which almost 2 million were never smokers of combustible cigarettes [2]. E-cigarettes have diverse designs but each device consists of a similar functioning unit. These vaporized liquid nicotine-delivering devices are composed of a nicotine cartridge, a vaporization chamber, and a rechargeable lithium battery [3]. Acute exposure studies have shown e-cigarettes to be associated with peripheral airway flow resistance, bronchial hyperreactivity and airway inflammation [4].

According to the National Academies of Science, Engineering, and Medicine report on the public health consequences of e-cigarettes, there is moderate evidence for increased cough and wheeze in adolescents who use e-cigarettes and an association with e-cigarettes and an increase in asthma exacerbations [5, 6]. The report also concludes that there is limited evidence for improvement in lung function and respiratory symptoms among adult smokers with asthma who switch to e-cigarettes completely or in part (dual use) [5, 6]. Prior studies among young adults have demonstrated associations between current e-cigarette use and asthma [7, 8]. Other studies have suggested that e-cigarettes may lead to overall improvement in subjective and objective respiratory outcomes [9, 10]. All studies on health effects of e-cigarette use have been limited by confounding by combustible cigarette smoking. Even studies that adjust for smoking in their models are subjected to residual cofounding. Additionally, there has been no large, nationally representative study on the association between e-cigarette use and asthma among never smoking adults. Therefore, we studied the association between e-cigarette use and self-reported asthma among never combustible cigarette smokers using the largest and most contemporary U.S. survey of e-cigarette use to date.

## Methods

Using 2016 and 2017 data from the Behavioral Risk Factor Surveillance System (BRFSS), a large cross-sectional telephone survey of adults aged 18 years and older in the U.S. [11], we studied a total of 402,822 self-reported never combustible cigarette smokers (defined as lifetime smoking < 100 cigarettes). The median survey response rate for all states, territories and Washington, DC, in 2016 was 47.0%, ranging from 30.7 to 65.0%. In 2017 the response rate was 45.1%, ranging from 30.6 to 64.1% [12, 13].

### Study measures

We only included participants who responded “no” to “*Have you smoked at least 100 cigarettes in your entire life*?” and classified them as never combustible cigarette smokers.

Participants were asked: “*Have you ever used an e-cigarette or other electronic ‘vaping’ product, even just one time, in your entire life*?” Participants who answered *“yes”* were then asked: “*Do you now use e-cigarettes or other electronic ‘vaping’ products every day, some days, or not at all*?” Participants who responded *“no”* to the first question were categorized as never e-cigarette users. Those who reported using e-cigarettes or other electronic ‘vaping’ products “some days” or “every day” were classified as occasional or daily e-cigarette users respectively. Current e-cigarette use, further classified as daily or occasional use, was the primary exposure.

Participants were asked***:*** *“Has a doctor, nurse, or other health professional ever told you that you have asthma,”* This was followed by the question *“Do you still have asthma?”* Only participants who answered in the affirmative to the first question and additionally reported current asthma were classified as having self-reported asthma, the primary outcome.

Age, sex, race, income, level of education and body mass index were self-reported. The income variable was further adjusted using the Federal Poverty levels in the United States for 2016 and 2017 [14]. The income variable incorporated the number of adults and children in a household as well as state of residence to categorize participants into those below the 100% federal poverty level, between 100 and 200% and above 200% federal poverty level.

### Statistical analysis

Details of the weighting methodology for BRFSS, which ensures national representativeness, are available elsewhere [1, 15]. Multivariable logistic regression models were used to assess the cross-sectional association between current e-cigarette use and self-reported asthma. The models were adjusted for age, sex, race, income, level of education and body mass index. Additionally, we run sensitivity analyses models after constructing 1:1 matching on a propensity score composed of age, sex and race. We expanded our analysis by including age, sex and race matched; 18–24 year old combustible cigarette smokers in the BRFSS.

## Results

Of 402,822 never combustible cigarette smokers, there were 3103 (0.8%) current e-cigarette users and 34,074 (8.5%) with self-reported asthma (Table 1). The median age group of current e-cigarette users was 18–24 years. Current e-cigarette users were more likely to be men, white, and below the 100% federal poverty line compared to never e-cigarette users.

**Table 1:** Baseline characteristics of the study population by e-cigarette use categories

	BRFSS 2016 & 2017	
	*N* = 402,822	
	E-cigarette use status	
Variables	Current (*N* = 3103)	Never (*N* = 399,719)
Median age group, years	18–24	45–49
Women (%)	32.8	56.2
Race		
White, (%)	57.2	59.6
Black, (%)	12.1	12.1
Asian, (%)	7.1	7.2
Hispanic, (%)	18.9	18.5
Others, (%)	4.7	2.6
Education		
Less than high school diploma, (%)	9.9	11.3
High School diploma, (%)	38.8	24.7
Some college, (%)	51.3	64.0
Federal poverty line		
< 100%, (%)	16.2	13.2
100–200%, (%)	19.0	18.8
> 200%, (%)	64.8	68.0
Body Mass Index (BMI)		
< 18.5 kg/m^2^, (%)	3.8	1.9
18.5 to < 25 kg/m^2^, (%)	44.5	33.7
25 to < 30 kg/m^2^, (%)	30.7	35.1
≥ 30 kg/m^2^, (%)	21.0	29.3
Asthma, (%)	10.8	8.1

**Fig. 1:**
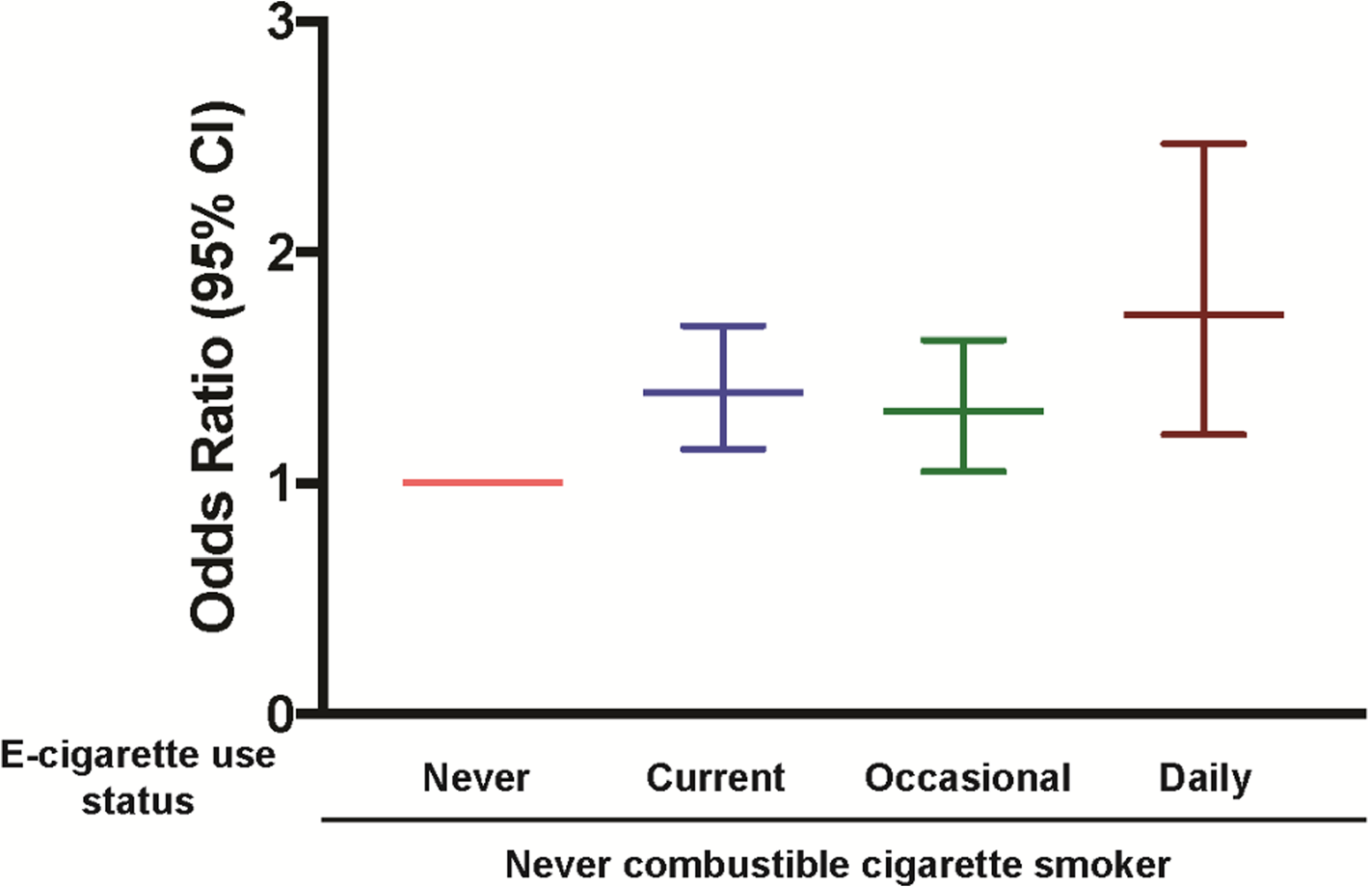
Association between e-cigarette use frequency and asthma among never combustible cigarette smokers

Current e-cigarette use was associated with 39% higher odds of self-reported asthma (Fig. 1) compared to never e-cigarette users (Odds Ratio [OR], 1.39; 95% confidence interval: 1.15, 1.68). The association of e-cigarette use with asthma was graded with increasing frequency of e-cigarette use. The odds ratio of self-reported asthma increased from 1.31 (95% confidence interval: 1.05, 1.62) in occasional users to 1.73 (95% confidence interval: 1.21, 2.48) in daily e-cigarette users, compared to never e-cigarette users. Similar results were observed with propensity score models.

## Discussion

In a nationally-representative sample of never smokers, we report significantly higher odds of asthma among current e-cigarette users compared to never e-cigarette users. E-cigarettes have been promoted as a less harmful alternative to combustible cigarettes and may play a role in smoking cessation [1, 16]. Study of the health effects of e-cigarettes has been limited by the relatively short time e-cigarettes have been on the market. Some studies have reported e-cigarette related acute toxicity reflected by increased airway resistance, oxidative stress and inflammatory responses [4, 17, 18], with reported cases of acute respiratory distress after e-cigarette use [19]. However, other acute exposure studies have reported no or minimal changes in respiratory symptoms, lung function or inflammatory markers with acute e-cigarette exposures, highlighting current controversy in the field [20–22].

To minimize confounding by combustible cigarette smoking use, we limited our analysis to never combustible cigarette smokers [23]. In a similar analysis, Schweitzer et al. demonstrated that current e-cigarette use was associated with currently having asthma (adjusted odds ratio [aOR], 1.48; 95% confidence interval: 1.26, 1.74) among Hawaii youth, independent of smoking and marijuana use [7]. However, these results may be subject to residual confounding. Our study reports similar point estimates (Odds Ratio [OR], 1.39; 95% confidence interval: 1.15, 1.68) in a much larger and more demographically geographically diverse population, and additionally adds the novel finding of a graded association with asthma with increasing frequency of e-cigarette use among adults. These estimates can be compared to findings from cross-sectional studies on the association between traditional cigarettes and asthma. Traditional cigarette smoking has been strongly associated with onset of asthma among nonatopic individuals (OR, 5.7: 95% confidence interval: 1.7, 19.2) [24].

Our findings have potential public health implications considering that 60% of e-cigarette users without a history of combustible cigarette smoking are younger than 25 years [2]. It is important to confirm our findings in a controlled study in which asthma and e-cigarette use are objectively characterized through airway hyper-responsiveness and lung function assessment.

In interpreting our hypothesis-generating findings, it is important to consider other arguments that may be of concern in this rapidly evolving field of e-cigarettes. For example, while it is known that asthma development may result after prolonged exposure to environmental irritants, many of the BRFSS study respondents may not have used e-cigarettes for extended time periods, thus challenging the plausibility of e-cigarettes leading to disease in a very short period of time. However, some studies have reported associations between e-cigarette exposure, asthma symptoms, and asthma exacerbations in susceptible individuals [8, 25]. E-cigarette vapor may also serve as a non-selective trigger unmasking an underlying subclinical asthma.

It is also useful to frame the biologic plausibility of our results in the context of what is known about smoking and asthma. A study on ten-year prevalence trends in respiratory symptoms and asthma in relation to smoking showed a high prevalence of physician-diagnosed asthma despite a decline in traditional smoking over the same period [26]. In addition, some countries with extremely high smoking prevalence such as Russia [27] do not have much higher asthma prevalence compared to other countries in their respective regions [28]. Also, asthma epidemics are yet to be reported in countries with high prevalence of e-cigarette use in recent years [29]. These arguments highlight current controversies in the field, supporting further biologic plausibility studies and longitudinal studies to assess the population-level and long-term health impact of these novel tobacco products.

Our study has notable limitations. The exposures and outcomes were self-reported, and there is no data on e-cigarette use initiation, duration, intensity (puffs/day) and flavorings used. Studies on the long-term respiratory health effects of e-cigarettes have been limited because these novel tobacco products have been on the market for a relatively shorter time. However, it is important to determine if e-cigarette using young adults with asthma may be at increased risk for exacerbation of asthma symptoms with rapid deterioration of lung function [6]. Importantly, the association of e-cigarette use and respiratory outcomes needs to be explored through longitudinal studies. Data on some key asthma confounders such as family or personal history of allergy (such as atopic dermatitis and allergic rhinitis), as well as second hand exposure to smoking were not available in this study and as such the possibility of residual confounding due to these variables cannot be ruled out.

Also, due to the cross-sectional nature of our study, we cannot infer causality. Additionally, considering that the age range of the study respondents who are current e-cigarette users is 18–24 years and that asthma prevalence is higher mostly during childhood [30, 31], it reasonable to argue that for some individuals e-cigarette use may have started after asthma diagnosis, discounting causality. However, e-cigarette use may be associated with acute exacerbations of respiratory symptoms.

The possibility of a self-selection based on a pre-existing condition cannot be discounted because individuals with a prior asthma diagnosis might avoid taking up smoking and self-select to e-cigarette use instead, which may be perceived as a less harmful nicotine containing product for their disease. Also, it is possible that report of cough/wheeze associated with vaping may be erroneously self-reported as a diagnosis of asthma [32] as it is known that inhalation of Propylene Glycol (PG) / Vegetable Glycerin (VG) mixtures can cause irritation and trigger the physiological reflex of cough/wheeze. To minimize this confusion, participants in this study were asked to self-report only diagnoses of asthma made from a doctor, nurse, or other health professional. Also e-cigarette vapor may serve as a non-selective trigger unmasking an underlying subclinical asthma. Despite the limitations, BRFSS provides a large sample size to study e-cigarette use specifically among this unique population of never combustible e-cigarette users.

## Conclusions

In conclusion, our findings from a large, nationally representative survey suggest increased odds of asthma among never combustible smoking e-cigarette users. This may have potential public health implications, providing a strong rationale to support future longitudinal studies of pulmonary health in young e-cigarette using adult.

## Data Availability

The datasets analyzed during the current study are publicly available in the Centers for Disease Control and Prevention (CDC) repository, [https://www.cdc.gov/brfss/annual_data/annual_2017.html].

## Abbreviations

BRFSS: Behavioral Risk Factor Surveillance System
PG: Propylene Glycol
VG: Vegetable Glycerin
